# Complete Genome Sequences of One Prodigiosin-Producing *Serratia marcescens* Strain ZPG19

**DOI:** 10.3389/fbioe.2021.665077

**Published:** 2021-05-11

**Authors:** Xue Li, Xinfeng Tan, Jing Zhang, Jie Zhang

**Affiliations:** State Key Laboratory of Biobased Material and Green Papermaking, School of Bioengineering, Qilu University of Technology (Shandong Academy of Sciences), Jinan, China

**Keywords:** *Serratia marcescens*, strain ZPG19, prodigiosin, genomic sequence, comparative genomics

## Introduction

As the typical species of the genus *Serratia, Serratia marcescens* is a rod-shaped, facultatively anaerobic, Gram-negative bacterium of the family Enterobacteriaceae (Hejazi and Falkiner, 1997; Matsushita et al., 2009; Gaultier et al., 2018). It has been reported as an opportunistic human pathogen that may cause the hospital-acquired infections (Ferreira et al., 2020). Remarkably, *S. marcescens* has the ability to produce a series of valuable products, including prodigiosin, chitinase, protease, lipase, nuclease, bacteriocin, surfactant, and wetting agent. The prodigiosin is a bioactive secondary metabolite with many pharmaceutical values such as antimicrobial, algicidal, anticancer, antimalarial, anti-inflammatory, anti-diabetic, and immunomodulatory effects (Atsushi et al., 2014; Darshan and Manonmani, 2015; Arivizhivendhan et al., 2018). Although it could be produced by several bacterial species from the genera *Serratia, Pseudoalteromonas, Vibrio*, and so on (Lee et al., 2011; Elkenawy et al., 2017), the genus *Serratia* is well-known as the main prodigiosin producing strains (Li et al., 2015).

Considering the potential applications, *S. marcescens* has attracted great attentions from many researchers Due to advances in high-throughput sequencing technologies, more and more sequencing projects have been set up and researchers could better understand the function, environmental adaptation and potential application of bacteria. There have been 682 genomes of *S. marcescens* reported in NCBI (https://www.ncbi.nlm.nih.gov/genome/?term=Serratiamarcescens). However, most of these strains were isolated from the intestines and ecological niches, such as air, soil, water, plants and animals. Here, we firstly isolated strain ZPG19 from the compost generated by aerobic composting of *Flammulina velutipes* residue collected in Dezhou, Shandong Province, China (37.45°N, 116.37°E). We sequenced and characterized its complete genomes in order to provide a promising resource to conduct the biosynthesis analysis and the molecular investigations of genus *Serratia*.

## Materials and Methods

### Genomic DNA Isolation

Strain ZPG19 of *S. marcescens* was cultured for 24 h in LB media (Tryptone 10 g/L, Yeast extract 5 g/L, NaCl10 g/L). Then genomic DNA was extracted with TIANamp Bacteria DNA Kit (TIANGEN Biotech Co., Ltd, Beijing, China) following the manufacturer's instructions. The quality and quantity of purified genomic DNA were determined by NanoDrop 2000 (Thermo Scientific, MA, USA).

### Genome Sequencing, Assembly, and Annotation

The genome of the strain ZPG19 was sequenced at Personal (Shanghai Personal Biotechnology Co., Ltd, China) using two different technologies: Illumina NovaSeq with 400 bp library and the PacBio Sequel with a 20-kb library. The adapters of the 3' end were removed using AdapterRemoval (Schubert et al., 2016). Raw reads were quality filtered and error corrected with SOAPEC (kmer = 17) (Luo et al., 2012). *De novo* assembly of the read sequences was carried out using the hierarchical genome assembly process workflow (Chin et al., 2016). The annotation of the sequences was carried out using a modified version of the Prokka annotation pipeline, which incorporated Prodigal 2.60, Aragorn, and RNAmmer 1.2 for the prediction of open reading frames, tRNAs, and rRNAs, respectively (Seemann, 2014; Yabe and Fukushima, 2020). The prediction of other ncRNAs was mainly obtained by comparing with Rfam database (Kalfari et al., 2018).

### Genome Comparison

We selected the whole sequenced genomes of five *S. marcescens* strains isolated from different habitats for comparative genomic analysis. Chromosomal genome comparison among strain ZPG19 and these five fully sequenced genomes was carried out by using progressive Mauve genome aligner with Geneious software at the default settings (Kearse et al., 2012).

### Direct Link to Deposited Data and Information to Users

The BioProject designations for *S. marcescens* strain ZPG19 are PRJNA665610. And the raw sequences have been deposited in GenBank under the accession numbers SRR12714697 in September 2020. Strain ZPG19 of *S. marcescens* is available from the China Center for Type Culture Collection (CTCC) under accession numbers M2019645.

## Interpretation of Data Set

### General Genome Features

We obtained 8,126,164 raw reads covering a total of 1,212,079,667 bp with 230× genome coverage for strain ZPG19. The complete chromosome contained a circular molecule of 5,269,270 bp with 59.49% G+C content. A total of 5,169 genes were predicted including 4,934 coding DNA sequences (CDSs), 95 tRNA genes, 22 rRNA genes and 118 other non-coding RNA genes. In this work, no plasmid was found. The detailed genomic information of ZPG19 and five *S. marcescens* strains was list in Table 1.

**Table 1:** Detailed information of Chromosomal genomes from *S. marcescens* Strain ZPG19 and five reported *S. marcescens* strains.

**Features**	**ZPG19**	**SM39**	**Sma274**	**SGAir0764**	**Db11**	**FS14**
Site of isolation	Compost	Blood	lab Stock	Air	Drosophila	Plant
No. of chromosome	1	1	1	1	1	1
No. of plasmid	0	2	1	1	0	0
Size (bp)	5,269,270	5,225,577	5,148,533	5,142,714	5,113,802	5,249,875
G + C (%)	59.49	59.82	59.53	59.54	59.51	59.47
Total genes	5,169	5,095	4,895	4,981	4,850	4,873
CDS	4,934	4,975	4,758	4,856	4,724	4,761
tRNA	95	88	95	91	88	91
rRNA	22	22	22	22	22	21
Other ncRNA	118	10	20	12	16	10
Reference	This work	Atsushi et al., 2014	Yabe and Fukushima, 2020	Gaultier et al., 2018	Li et al., 2015	Li et al., 2015

### Genome Comparison

The whole-genome sizes, GC contents and gene contents of the six *Serratia* strains were comparable with a slight difference (Table 1). At the same time, the number of genes increased with the enlargement of genome size. The number of plasmids varied in six *Serratia* strains. SM39 carried two plasmids, Sma274 and SGAir0764 carried one plasmid, respectively. While the other three *Serratia* strains analyzed had no plasmid.

**Figure 1:**
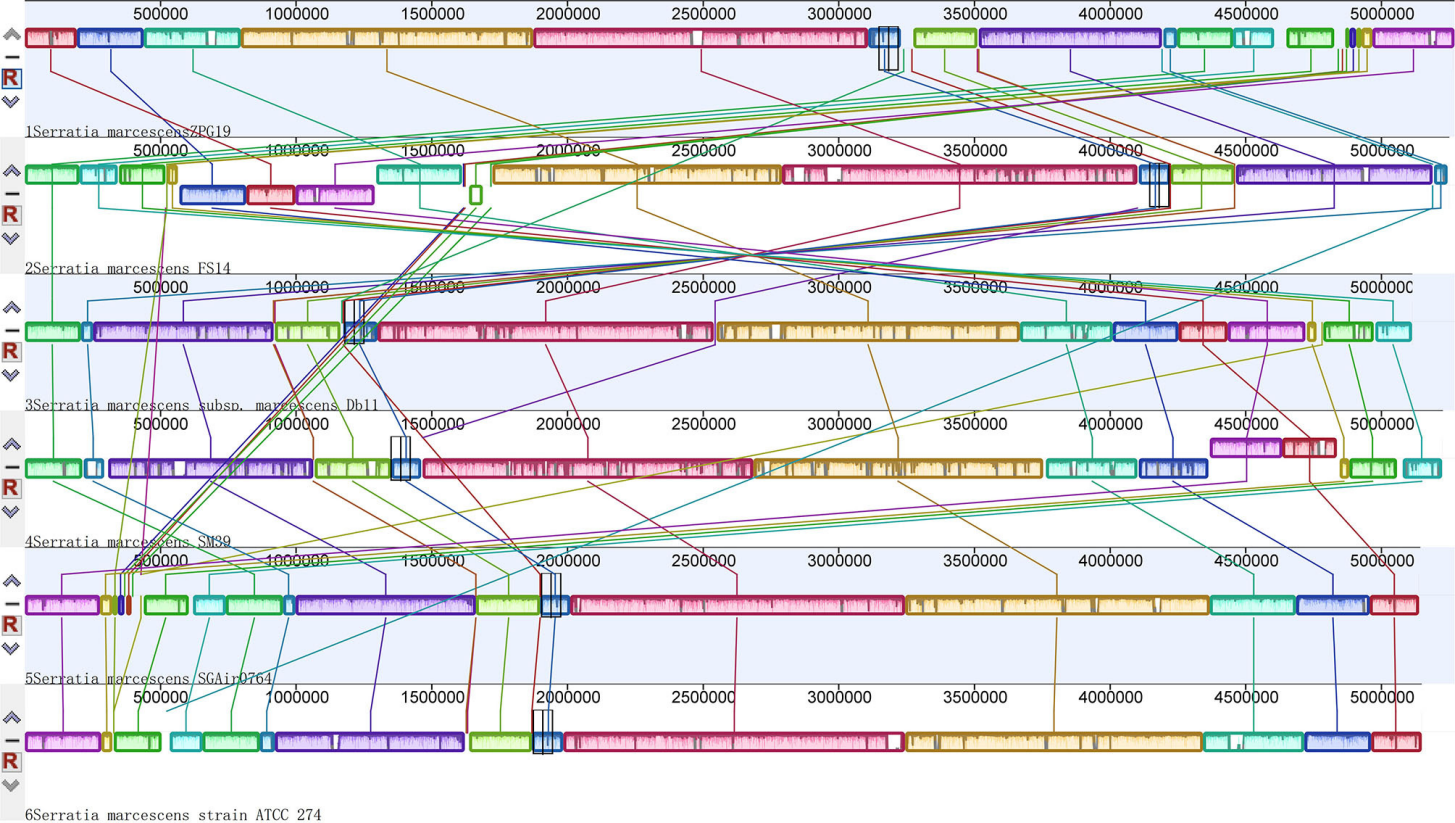
Global multiplealignments of chromosomes from six Serratia strains using progressive MAUVE with default parameters. Colored blocks indicated the genome sequences that aligned to part of another genome and was possibly homologous and internally free from genomic rearrangement (locally collinear blocks). White regions represented sequences that did not aligned and probably contained sequences specific to a particular genome. Blocks below the center line showed regions that aligned in the reverse orientation. The names of the strains were listed at the bottom of the blocks.

A global alignment of genome sequences from six *Serratia* strains was performed using Mauve software and the results showed that the synteny between *S. marcescens* was not very conserved (Figure 1). Gene rearrangements were commonly observed along the whole stretch of the six chromosomal genomes which was consistent with previous reports (Li et al., 2015).

### Prodigiosin-Producing Enzymes

**Figure 2:**
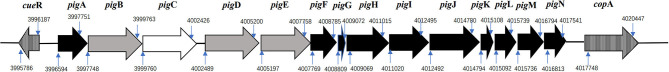
The *pig* cluster of prodigiosin biosynthesis in strain ZPG19. Genes were symbolized by arrows. The white arrows denoted genes involving in condensing enzymes; black arrows represented genes encoding proteins required for the biosynthesis of the monopyrroles; gray arrows genes encoding proteins required for the biosynthesis of 4-methoxy-2,2-bipyrrole-5-carboxyaldehyde; vertical striped arrows denoted flanking genes *cue*R and *cop*A; The numbers in small blue arrows indicated the start positions and end positions of each gene.

We have confirmed that the strain ZPG19 could produce prodigiosin via HPLC-MS method. Here, the prodigiosin-producing inventory of ZPG19 was identified including genes for 3-oxoacyl-[acyl-carrier protein] reductase (*fab*G), [acyl-carrier-protein] S-malonyltransferase (*fab*D) and *pig* cluster. The *pig* cluster contained 14 candidate genes which arranged *pig*A through to *pig*N flanked by *cue*R and *cop*A (Figure 2). We have putatively assigned the products of one gene to the condensing enzymes. Ten genes that encoded proteins required for the biosynthesis of the monopyrroles. Three genes encoding proteins required for the biosynthesis of 4-methoxy-2,2-bipyrrole-5-carboxyaldehyde. This observation is congruent with *S. marcescens* strain FS14 and *S. plymuthica* strain AS13 in earlier studies (Li et al., 2015). The order of the genes was conserved among these *Serratia* species and the corresponding 14 predicted proteins were similar in size and share significant amino acid.

In conclusion, the complete genome of *S. marcescens* strain ZPG19 was sequenced and assembled into one chromosome. Comparative genome and sequence analyses showed that rearrangements occurred in six *Serratia* strains. Genes *fab*G, *fab*D, and *pig* cluster responsible for prodigiosin production were detected in this work.

## Data Availability Statement

The datasets presented in this study can be found in online repositories. The names of the repository/repositories and accession number(s) can be found in the article/Supplementary Material.

## Author Contributions

Wet lab execution was performed by XL. Data processing and handling was performed by XT. The manuscript was written by JingZ. Project planning was conducted by JieZ. All authors contributed to the article and approved the submitted version.

## Conflict of Interest

The authors declare that the research was conducted in the absence of any commercial or financial relationships that could be construed as a potential conflict of interest.
